# Seasonal Variation and Crop Sequences Shape the Structure of Bacterial Communities in Cysts of Soybean Cyst Nematode

**DOI:** 10.3389/fmicb.2019.02671

**Published:** 2019-11-21

**Authors:** Weiming Hu, Noah Bernard Strom, Deepak Haarith, Senyu Chen, Kathryn E. Bushley

**Affiliations:** ^1^Department of Plant and Microbial Biology, University of Minnesota, Saint Paul, MN, United States; ^2^Department of Entomology and Nematology, University of Florida, Gainesville, FL, United States; ^3^Department of Plant Pathology, University of Minnesota, Saint Paul, MN, United States; ^4^Southern Research and Outreach Center, University of Minnesota, Waseca, MN, United States

**Keywords:** bacterial community, biological control, crop rotation, metabarcoding, soybean cyst nematode, *Heterodera glycines*

## Abstract

Soybean cyst nematode (SCN), *Heterodera glycines* Ichinohe, is the number 1 pathogen of the important economic crop soybean. Bacteria represent potential biocontrol agents of the SCN, but few studies have characterized the dynamics of bacterial communities associated with cysts under different crop rotation sequences. The bacterial communities in SCN cysts in a long-term soybean–corn crop rotation experiment were investigated over 2 years. The crop sequences included long-term soybean monoculture (Ss), years 1–5 of soybean following 5 years corn (S1–S5), years 1 and 2 of corn following 5 years soybean (C1 and C2), and soybean–corn annual rotation (Sa and Ca). The bacterial 16S rRNA V4 region was amplified from DNA isolated from SCN cysts collected in spring at planting, midseason (2 months later), and fall at harvest and sequenced on the Illumina MiSeq platform. The SCN cyst microbiome was dominated by Proteobacteria followed by Actinobacteria, Bacteroidetes, and Verrucomicrobia. The bacterial community composition was influenced by both crop sequence and season. Although differences by crop sequence were not significant in the spring of each year, bacterial communities in cysts from annual rotation (Sa and Ca) or crop sequences of early years of monoculture following a 5-year rotation of the alternate crop (S1 and C1) became rapidly differentiated by crop over a single growing season. In the fall, genera of cyst bacteria associated with soybean crop sequences included *Rhizobacter*, *Leptothrix*, *Cytophaga*, *Chitinophaga*, *Niastella*, *Streptomyces*, and *Halangium*. The discovery of diverse bacterial taxa in SCN cysts and their dynamics across crop rotation sequences provides invaluable information for future development of biological control of the SCN.

## Introduction

Soybean [*Glycine max* (L.) Merr.] is an important crop worldwide, and the Midwestern region of the United States, including states such as Illinois, Iowa, and Minnesota, produces over 80% of total soybean in the United States (Hartman et al., 2011). The soybean cyst nematode (SCN), *Heterodera glycines* Ichinohe, is the primary yield-limiting factor in those regions (Wrather and Koenning, 2006). Planting resistant cultivars have historically been one of the primary approaches for managing the SCN (Chen et al., 2001). However, most of the resistant cultivars are developed from a single source of resistance (PI8898) and there is evidence and significant risk that SCN in the field is already breaking this resistance over years of continuous growth of resistant soybean (Koenning, 2004). Nematicides, which were also commonly used for control of plant-parasitic nematodes in the past, are not cost-effective for managing SCN (Chen et al., 2001), and many have been banned as they pose serious risks to both human health and the environment (Mereu and Chapman, 2010; UNEP, 2015). Crop rotation with a non-host crop such as corn (*Zea mays* L.) is an alternative approach. In Minnesota, a combination of annual rotation of resistant soybean with corn is the most common rotation. The annual corn rotation has some effectiveness in managing the SCN, but it may take up to 5 years of corn rotation to reduce the SCN population density to a level that does not cause significant damage to a susceptible soybean in northern climates (Chen et al., 2001). Biological control has been explored, and offers an attractive and environmentally sustainable alternative for SCN control as part of an integrated management plan (Chen and Dickson, 2012).

The effects of crop rotation on SCN populations and crop yield have been extensively studied in different soybean production regions (Ross, 1962; Crookston et al., 1991). The yield of both rotation crops was shown to increase in the first year after rotation (Howard et al., 1998). Crop rotation has also been found to influence soil microbial communities. Early research found that the total microbial community diversity in soil was greater in a wheat-soybean rotation than under monoculture (Lupwayi et al., 1998; Perez-Brandan et al., 2014). In a corn-soybean rotation system, it was reported that crop rotation affected the total microbial community based on phospholipid fatty acid analysis (PLFA; Vargas Gil et al., 2011). Furthermore, differences in the bacterial community composition were detected by denaturing gradient gel electrophoresis (DGGE), but this method was not sufficient to fully capture the diversity and richness of the community (Yin et al., 2003). More recently, metabarcoding studies have shown that the bacterial communities in soil may shift in response to tillage and crop rotation regimes (Yin et al., 2010).

Each growing season, under SCN susceptible soybean, the SCN produces abundant cysts, each containing hundreds of nematode eggs, which form a resistant overwintering structure that can protect the viability of nematode eggs in soil for up to a decade (Koenning, 2004; Chen, 2011). Thus, understanding the survival and dynamics of bacteria inhabiting the microenvironment of SCN cysts in agroecosystems is of ultimate importance for discovering new and effective biological control agents. However, there is very limited knowledge of how bacterial communities within SCN cysts are affected by crop rotation. Previous research has shown that the length of soybean monoculture impacted the bacterial communities inside SCN cysts, as distinct bacterial communities and taxonomic groups were found in cysts from short term (< 8 years) versus longer term (> 8 years) soybean monoculture (Zhu et al., 2013). A recent study demonstrated that the fungal communities in SCN cysts were also affected by crop rotation (Hu et al., 2018). With the emergence of metabarcoding approaching using high-throughput sequencing, there is growing demand and opportunity to explore the diversity and dynamics of microbial communities associated with the SCN cysts in agroecosystems in order to facilitate SCN management using biological control.

The bacteria found in SCN cysts have been shown to be highly diverse (Nour et al., 2003), and hundreds of bacterial species have been isolated from the cysts of the SCN. The dominant bacterial genera isolated from cysts from a field in southern Ontario, Canada, were *Lysobacter* spp. and *Variovorax* spp. (Nour et al., 2003). In addition, taxa belonging to Proteobacteria (Alpha, Beta, Delta, and Gamma-Proteobacteria) and Bacteroidetes were the dominant phyla detected by the DGGE method used in another study (Yin et al., 2003). Several sulfate-reducing bacteria and Actinobacteria in the genera *Actinomadura* and *Streptomyces* were also detected. Similarly, a greenhouse study of bacterial communities found in cysts grown in a long-term SCN suppressive soybean monoculture soil found that Proteobacteria, Actinobacteria, Bacteroidetes, and Firmicutes were among the most abundant bacterial phyla present in SCN cysts (Hussain et al., 2018).

Bacteria isolated from cysts may have potential for control of SCN. Several species have been shown to inhibit egg hatch and to control the SCN in both *in vitro* and greenhouse assays (Bent et al., 2006). Rhizobacteria isolated from soybean roots, for example, reduced SCN egg hatch in the greenhouse, but gave inconsistent results in field trials (Tian and Riggs, 2000). *Bacillus* and *Pseudomonas* species have been shown to reduce SCN cyst numbers (Kloepper et al., 1992) and thus likely inhibit reproduction within soybean roots. The well-known biological control bacterium *Pasteuria nishizawae*, which is an obligate parasite of nematodes, was isolated from second-stage juvenile (J2) of SCN (Noel and Stanger, 1994). Although *Pasteuria* has been extensively studied in interactions with the root-knot nematode, there are only a few additional reports of its isolation from SCN in Minnesota (Chen and Dickson, 2012). Toxins or antibiotics produced by bacteria are in commercial use as nematode biopesticide products. Avermectins, produced by *Streptomyces avermitilis*, for example, can kill nematodes (Egerton et al., 1979) and form the active ingredient of the seed treatment Abermectin^TM^ for SCN. Other known producers of antihelmenthic compounds include *Pseudomonas fluorescens*, *Bacillus chitinosporus*, and *Bacillus firmus* (Chen and Dickson, 2012). As the bacterial taxa within cysts have not been well characterized, it is likely that other bacterial taxa effective in reducing SCN populations remain to be discovered.

How crop sequences impact the microbial communities within the SCN cysts is also important because crop rotation is a common cultural practice for managing SCN. In order to investigate how crop rotation sequences influence the bacterial communities in cysts, we conducted research in a long-term field experiment that has been in soybean–corn rotation for over 30 years. Previous research at this site suggested that the soil ecology under corn and soybean crop rotation sequences were different based on changes in the nematode community (Grabau and Chen, 2016b). Moreover, among the nematodes that could affect soybean yield, the SCN was the most important at this site, and SCN density was shown to increase with increasing years of soybean monoculture over 5 years (Grabau and Chen, 2016a). In a previous companion study analyzing fungi associated with the same SCN cysts analyzed in this study, fungal communities were shown to vary by crop sequence as well as over the crop growing season (Hu et al., 2018). This study seeks to (1) characterize the key bacterial groups associated with the cysts of SCN in a soybean–corn rotation system, (2) to understand the influence of seasonal variation and crop rotation on these communities, and (3) to identify taxa enriched and associated with soybean crop sequences with potential for biological control.

## Materials and Methods

### Field Experimental Design and Management

This study was performed in a field site (44°04′N, 93°33′W) at the University of Minnesota Southern Research and Outreach Center in Waseca, MN, United States. A corn–soybean crop rotation sequence was initiated in 1982. The experimental design of this field is detailed in Grabau and Chen (2016a). The factor of cropping sequence effects was examined in a complete randomized block design with four replicate plots. Each plot had six rows of crops.

This study focused on a subset of the corn-soybean crop rotation sequences in 2015 and 2016, sampled at three time points each season: spring (mid May), midseason (late July to early August), and fall (mid-October) (Hu et al., 2018). The 10 corn-soybean rotation treatments were: (1) continuous soybean monoculture since 1982 (Ss), (2) corn and soybean annual rotation (Ca, Sa), (3) first and second year corn (C1, C2) after 5 years monoculture of soybean, and (4) first through fifth year soybean (S1, S2, S3, S4, S5) after 5 years of corn monoculture, with the C indicating corn and the S soybean sequences. Pioneer P91Y90 (SCN-susceptible soybean) and DKC50-82 RIB (BT corn) were used for all soybean or corn plots. The plots were plowed with a field chisel after harvest at fall and before planting at spring each year. Nitrogen was only applied to the corn plots at a rate of 224.4 kg/ha. The herbicide glyphosate (Roundup) was applied for weed control. The insecticide Endigo (active ingredient: thiamethoxam and lambda-cyhalothrinm) was sprayed at 245 g/ha at midseason each year.

### Cyst Sampling and Collection

Cyst sampling and collection were performed as detailed in Hu et al. (2018). Briefly, sucrose flotation and centrifugation method (Jenkins, 1964) was used to extract cysts from about 4 kg soil per plot. From each plot, six soil samples were collected with a shovel at a depth of 20 cm within 4 cm of plant growth in the center two rows and soaked in water prior to extracting cysts. A total of 50 individual intact mature (brown) SCN cysts were picked under an inverted microscope from each plot, surface-sterilized with 0.5% NaOCl for 3 min, and rinsed thoroughly with autoclaved water. The cysts were then stored at −80°C until subsequent DNA extraction. An insufficient number of cysts were collected from the S1 of midseason 2015 and this treatment was thus omitted from midseason 2015 analyses.

### Cyst DNA Extraction and Metabarcoding

Surface-sterilized cysts were crushed in a small centrifuge tube using a pestle. DNA was isolated from the macerated cysts according to a modified CTAB protocol described in Hu et al. (2017). The DNA extracted from the 50 pooled cysts collected from each plot was used to construct a single metabarcode library, with four replicates per crop sequence at each collection time point. The PCR amplification, library preparation, and sequencing were conducted at the University of Minnesota Genomic Center, Saint Paul, MN, United States (Gohl et al., 2016). The universal bacterial primers targeting the 16S rRNA V4 region with primers 515F (GTGCCAGCMGCCGCGGTAA) and 806R (GGACTACHVGGGTWTCTAAT) were used. The Illumina index and flow cell adapters were amplified together with the V4 primers during the first step PCR, and dual-index barcode sequence was added at the second PCR step (Gohl et al., 2016). The samples from the same year were pooled and sequenced on one Illumina MiSeq lane with the 2 × 300 bp kit. For each paired-end lane of MiSeq, additional samples were included in order to assess the PCR and sequencing error in the downstream pipeline, including blank control samples, a mock community sample, which had equal amount of DNA isolated from pure bacterial cultures, and nine randomly chosen cyst samples for technical replicates.

### Sequence Quality Control and Processing

The platform yielded 24,703,544 sequences that passed quality control filters. The make.contigs step in Mothur v.1.39.5 (Schloss et al., 2009) was used to pair the Illumina forward and reverse reads, and those sequences having less than 150 bp overlap were removed. Any remaining sequences which had more than 2 bp mismatch of V4 region primers were also removed. Sequences that had more than eight homopolymers, ambiguous bases, or were outside of the 200–400 bp length range were filtered out. Sequences that did not align to the Silva-based bacterial V4 region were excluded. After the above filtering steps, the remaining high-quality sequences were then imported into QIIME V1.9.1 (Caporaso et al., 2010). The UCHIME (Edgar et al., 2011) pipeline was used to detect chimeras, and operational taxonomic units (OTUs; Blaxter et al., 2005) were picked utilizing a closed OTU picking approach with 97% similarity. Taxonomy was assigned to the OTU referred to the SILVA database (SILVA.123.1_SSURef_Nr99).

### Analysis of OTU Diversity and Relative Abundance

The OTU tables generated by QIIME were imported into R version 3.4.2 (R Core Team, 2014) for downstream analyses. The OTUs that had less than 10 total counts across all the samples or could not be assigned to the domain Bacteria were removed prior to downstream analyses. Overall, an average of 65,219 high-quality sequences were generated per sample, with the highest 129,573 per sample and the lowest 2,245 per sample. One sample that had an extremely low OTU count (294) was excluded from the analysis. The data were not rarefied and raw read counts were used after filtering with these quality control steps. Two measures of alpha-diversity, observed OTUs and Shannon alpha diversity index, were calculated using the package “Phyloseq” (McMurdie and Holmes, 2013) and plotted in R using “ggplot2” (Wickham, 2009).

For the statistical analysis of the alpha diversity indexes, the data were normally distributed and not transformed. An ANOVA model was constructed, which included crop rotation sequences, sampling time points, and replicates as independent variables together with the interaction between year, crop sequences, and Season (Y = Year + CropSequences + Season + Year^∗^CropSequences + Year^∗^Season + CropSequences^∗^Season + Year^∗^CropSequences ^∗^Year). Because in the full model we observed interactions between year, crop sequences, and seasons, another set of analyses were conducted in which year was not included in the model as a repeated measure. Thus, data were analyzed separately within each year using the model Y = CropSeq + Season + Replicates + CropSeq^∗^Season. This model was also used to test significant differences over sampling seasons within each year. Significant differences were reported at *P* < 0.05 throughout the manuscript, unless otherwise reported.

For beta diversity, the OTU counts were transformed to relative abundance of each sample and normalized by using a log_2_ transformation. A Bray–Curtis dissimilarity matrix was calculated using the R package “Vegan” (Oksanen et al., 2017), and the significance of Bray–Curtis dissimilarity matrix of cysts among all crop sequences, combined soybean vs. combined corn sequences, and among soybean sequences only was tested respectively by the Adonis function. The “Procrustes” function was used to detect changes in cyst bacterial communities over sampling time points and crop sequences.

In order to identify taxa specifically associated with cysts from crop sequences of a particular crop, several approaches were used. We analyzed the relative abundance of bacterial taxa at the Class and OTU levels using ANOVA. The relative abundance was transformed using log_2_ to improve the homogeneity. Additionally, a spearman rank correlation test was used to test for Classes and OTUs that were correlated with the increasing years of soybean monoculture (S1–S5). Finally, the linear discriminant analysis (LDA) effect size (LEfSe) algorithm (Segata et al., 2011) was used to detect the OTUs that differed in abundance in cysts when compared between corn and soybean crop sequences. This method identifies both the consistency of association and enrichment of specific taxa with each crop (e.g., soybean and corn) within the framework of the hierarchical phylogenetic relationships between taxa. For this analysis, the three corn sequences were pooled as the corn treatment and the seven soybean sequences were pooled together as the soybean treatment. LEfSe uses a non-parametric factorial Kruskal–Wallis (KW) sum-rank test between these two groups, corn and soybean crop sequences, and taxa that differ significantly between the crops are further tested using the (unpaired) Wilcoxon rank-sum test across different crop sequences within corn and soybean internally. At the last step, LEfSe uses LDA to estimate the effect size of each taxa, and significance at each taxonomic level (e.g., phylum, class, order, genus, species). Taxa enriched and associated with corn and soybean, respectively, at phylum, class, and order level were displayed in cladograms generated using the LEfSe tools in Galaxy (Segata et al., 2011).

## Results

### Taxonomic Composition of Bacteria in Cysts

**FIGURE 1:**
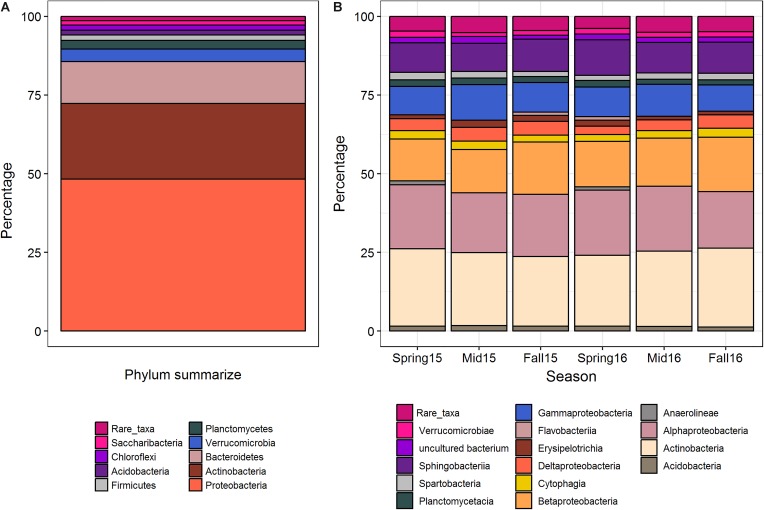
Summary of the average percentage of OTUs at the phylum level across all cyst samples **(A)**, and average percentages at the class level across seasons **(B)**. Rare taxa include those phyla or class that contributed less than 0.1% to the whole community.

**FIGURE 2:**
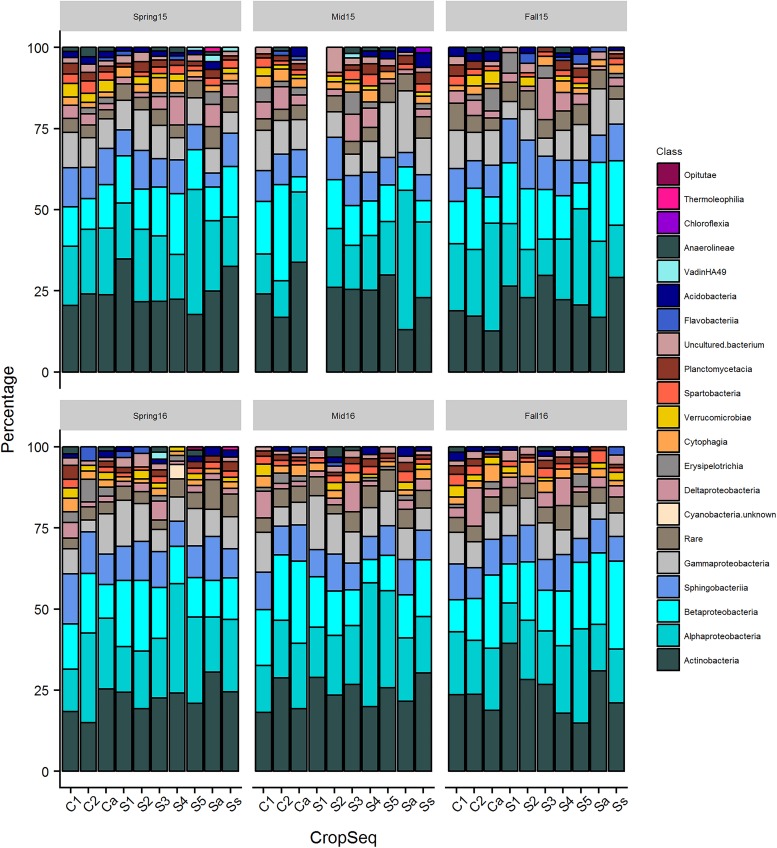
The distribution of bacterial classes containing at least 2% of total OTUs across crop sequences for each season. Percentage of OTUs from each class out of the total from the cyst community is shown on *y*-axis and taxa with higher percentage of OTUs are shown toward bottom of each column.

Overall, when averaged across crop sequences and seasons, the predominant bacterial phyla in cysts were Proteobacteria, which comprised nearly half (48.4%) of all taxa found in cysts. These were followed by Actinobacteria (24.0%), Bacteroidetes (13.3%), Verrucomicrobia (4.0%), Planctomycetes (2.7%), Firmicutes (1.7%), Acidobacteria (1.6%), Chloroflexi (1.6%), Saccharibacteria (1.5%), and 24 other phyla which had less than 1% relative abundance (Figure 1). At the class level, Actinobacteria (23.6%) was the most abundant class, and comprised nearly all of the phylum Actinobacteria (Figure 1). Among the Proteobacteria, Alphaproteobacteria (19.8%), Betaproteobacteria (15.1%), Sphingobacteria (9.62%), Gammaproteobacteria (9.6%), and Deltaprobacteria (3.8%) were the dominant classes (Figure 1). Other classes comprising greater than 2% of total OTUs included Erisypelotrichia, Cytophagia, Thermoleophilia, Planctomycetacia, Acidobacteria, Spartobacteria, Verrucomicrobiae, Acidibacteria, Flavobacteria, Anaerolineae, Chloroflexia, Opitutae, Gemmatinonadetes, Phycisphaerae, and the vadiniHA49 group (Figure 2). Genera containing the largest percentage of OTUs in cysts corresponded to *Streptomyces*, *Rhizobacter*, *Rhizobium*, *Aquincola*, *Actinocoralia*, *Bradyrhizobium*, *Niastella*, *Massalia*, *Lechevelaria*, and *Halangium* (Supplementary Figure S1).

### Variation in Abundance of Bacterial Taxa Across Season and Crop Sequences

Classes Actinobacteria, Acidimicrobiia, Alphaproteobacteria, Deltaproteobacteria, and Gammaproteobacteria differed significantly (*p* < 0.05) in relative abundance across different seasons when pooled across crop sequences (Figure 1 and Supplementary Table S1). A total of seventeen bacterial classes were found to differ significantly (*p* < 0.05) across crop sequences in at least four seasons (Supplementary Table S2). Among these, those that had the most significant (< 0.0001) differences in relative abundance across crop sequences in at least three seasons included Alphaproteobacteria, Betaproteobacteria, Planctomycetacia, Gemmatimonadetes, Acidimicrobiia, and Thermoleophilia (Supplementary Table S2). Among these, Alphaproteobacteria, Planctomycetacia, Gemmatimonadetes, and Thermoleophilia were generally more abundant in corn sequences, while Betaprotebacteria and Acidomicrobia were generally more abundant in soybean sequences (Supplementary Table S2). Other classes showing significant (*p* < 0.05) differences in relative abundance in at least three seasons included Holophagae, Thermomicrobia, Acidobacteria, KD4-96 group, Chloroflexia, Gammaproteobacteria, TK10 group, Clostridia, Phycisphaerae, Chlorobia, and Chlamydiae (Supplementary Table S2). However, no classes were found to significantly (*p* > 0.05) increase in relative abundance and positively correlate with increasing years of soybean monoculture in spearman rank correlation tests (data not shown).

Although 256 OTUs differed significantly (*p* < 0.05) in relative abundance across crop sequences (Supplementary Table S3), very few of these showed significant differences in more than a single season (Supplementary Table S3). Only 12 OTUs differed significantly across sequences in at least two seasons (Supplementary Table S3). One was identified to species, *Rickettsia felis* in Rickettsiales (Alphaproteobacteria), and several others could only be identified to genus, including *Kaistia* and *Bauldia* in Rhizobiales (Alphaproteobacteria), *Enterobacter* (Gammaproteobacteria), and *Longispora* (Actinobacteria), while the remaining belonged to the orders Acidobacteria Subgroup 17 (Acidobacteria), Chloroflexales (Chloroflexia), Rhizobiales (Alphaproteobacteria), Myxococcales (Deltaproteobacteria), the NKB5 group (Gammaproteobacteria), and Chthoniobacterales (Verrumicrobia) (Supplementary Table S3). A spearman rank correlation test identified six OTUs in fall of 2015 and five OTUs in spring of 2016 that were significantly correlated with increasing years of soybean monoculture (Supplementary Table S4). These included members of the Rhizobiales (Alphaproteobacteria) in families Bradyrhizobiaceae, Rhizobiaceae, and Hyphomicrobiaceae, two OTUs in Sphingobacteriales (Sphingobacteria) in family Chitinophagaceae, one in Nitrosomadales (Betaproteobacteria), one in Planctomycetales (Planctomycetacia) in Planctomycetaceae, one in genus *Solirubrobacter* (Thermoleophilia), and one in the genus *Verrucomicrobium* (Verrucomicrobia) (Supplementary Table S4). Surprisingly, most of these were negatively correlated with years of soybean monoculture year except for *Verrucomicrobium* (Supplementary Table S4).

### Alpha Diversity Affected by Seasons and Crop Sequences

Alpha diversity of bacterial communities was affected by both crop sequence and season in both years (Table 1). This effect of crop sequence and season was significant (*p* < 0.001) for observed species and Shannon index in 2015. Although the effect of crop sequence was marginally significant (*p* = 0.06) for the Shannon index in 2016, the effect of season was significant (*p* < 0.01) and an interaction was observed between crop sequences and season in 2016 (Table 1). However, observed OTUs did not show any significant effects for 2016 (Table 1). According to both observed OTUs and Shannon alpha diversity indexes, the bacterial community at midseason was less diverse than in spring or fall in 2015, whereas in 2016, only the Shannon index showed lower diversity at midseason compared to spring or fall (Table 1). Similarly, the soybean–corn crop sequences alone had a significant effect on the community diversity at spring and fall in both years, but not at midseason of either year (Table 2). In spring of 2015, C2 had the highest number of observed OTUs, while S1 had the lowest (Table 2). In fall of both years (Table 2), corn sequences (C1, C2, and Ca) tended to have a slightly higher diversity index (both observed OTUs and Shannon) than soybean crop sequences, although these differences were not significant between all corn and soybean crop sequences (Table 2). Overall, both season and crop sequences had some effect on the diversity of bacteria found in SCN cysts, with corn sequences generally having higher diversity compared to soybean sequences and diversity tending to be lower in midseason than in spring or fall of both years.

**TABLE 1:** Bacterial community alpha diversity indexes across season.

**Season**	**2015**		**2016**	
		
	**Observed**	**Shannon**	**Observed**	**Shannon**
Spring	1071 a	4.42 a	1030 a	4.61a
Mid	799 b	4.20 b	1039 a	4.26b
Fall	1052 a	4.49 a	1034 a	4.61a
**ANOVA:**				
CropSeq	<0.001^∗∗∗^	<0.001^∗∗∗^	0.86	0.060
Season	<0.001^∗∗∗^	<0.001^∗∗∗^	0.99	0.002^∗∗^
CropSeq^∗^season	0.14	0.4	0.52	0.006^∗∗^

*The data are means across all 40 samples, 10 crop sequences each with four replicates within each season. Significant differences are reported at (.) *P* < 0.1, (^∗^) *P* < 0.05, (^∗∗^) *P* < 0.01, and (^∗∗∗^) *P* < 0.001. Different letters in a column indicate a significant difference between seasons according to the Least Significant Difference (LSD) test at *P* < 0.05.*

**TABLE 2:** Bacterial community alpha diversity indices across crop sequences within each season.

	**2015 Spring**		**2015 Midseason**		**2015 Fall**		**2016 Spring**		**2016Midseason**		**2016Fall**	
						
**CropSeq**	**Observed**	**Shannon**	**Observed**	**Shannon**	**Observed**	**Shannon**	**Observed**	**Shannon**	**Observed**	**Shannon**	**Observed**	**Shannon**
C1	908bc	4.32a	762ab	4.37a	1264a	5.03a	798a	4.13d	1027a	4.43a	1024a	4.87ab
C2	1440a	4.76a	1269a	4.52a	1313a	4.9ab	1102a	4.53bcd	1164a	4.83a	1157a	4.99a
Ca	1101abc	4.34a	837ab	4.44a	1276a	4.9ab	940a	4.24cd	1072a	4.53a	1202a	4.97a
S1	683c	4.46a	NA	NA	993ab	4.24cd	1147a	5.1a	1126a	4.34a	916a	4.17e
S2	919bc	4.17a	592b	3.95a	1067ab	4.27cd	1004a	4.18d	1017a	4.07a	1001a	4.41de
S3	1021bc	4.23a	750ab	3.95a	986ab	4.34cd	947a	4.17d	1062a	4.22a	976a	4.51cd
S4	927bc	4.23a	751ab	4.02a	794b	4.2cd	1013a	4.3cd	908a	3.89a	850a	4.54bcd
S5	1152ab	4.45a	777ab	4.13a	758b	4.06d	1045a	4.52cd	762a	4.36a	945a	4.48cde
Sa	1285ab	4.6a	745ab	4.23a	1051ab	4.54bc	1082a	4.98ab	1144a	4.28a	929a	4.4de
Ss	1136ab	4.43a	708ab	3.97a	1021ab	4.39cd	1179a	4.64bc	762a	3.63a	1163a	4.77abc
*P*-value	0.003^∗∗^	0.18	0.06	0.48	0.01^∗^	<0.001^∗∗∗^	0.83	< 0.001^***^	0.85	0.43	0.02^∗^	< 0.001^***^

*Data were means of four replicates, significant values were reported at (^∗^) *P* < 0.05, (^∗∗^) *P* < 0.01, and (^∗∗∗^) *P* < 0.001. Different letters in a column indicate significant difference across crop sequences within each season at *P* < 0.05 according to LSD test.*

### Beta Diversity Affected by Crop Sequences and Seasons

Adonis analyses using a complete model (Bray–Curtis ∼ Season + CropSequence + Year + Season^∗^Year + Season^∗^Crop- Sequence + CropSequence^∗^Year + Season^∗^CropSequence^∗^Year) showed that crop sequence was the most significant (*p* < 0.0001) factor affecting community composition (Table 3). While neither season nor year was significant by themselves, the interactions between season and crop sequences and crop sequences and year were also significant (*p* < 0.0001) (Table 3). Because of the interactions between crop sequence and season, the Adonis analyses were also performed separately for each season and the crop sequences had a significant (*p* < 0.05) effect on the bacterial communities in cysts at all sampling seasons (Table 4). However, when all communities from corn or soybean crop sequences were pooled, the effects of crop species (e.g., soybean vs. corn) were observed only at midseason and fall in both years, but not in spring of either year (Table 4).

**TABLE 3:** Adonis test of effect of year, season, and crop sequence on Bray–Curtis distance index.

**Treatment**	***F***	***P*-value**
Season	1.1997	0.13
CropSeq	2.6219	< 0.0001^***^
Year	1.015	0.39
CropSeq^∗^Season	1.2882	< 0.0001^***^
Season^∗^Year	1.1101	0.23
CropSeq^∗^Year	1.4019	< 0.0001^***^
SeasonRep^∗^CropSeq^∗^Year	1.2301	< 0.0001^***^

*Significant *P* values were reported at (^∗^) *P* < 0.05 and (^∗∗∗^) *P* < 0.001.*

**TABLE 4:** *P*-value of Adonis test of Bray–Curtis dissimilarity matrix across crop sequence treatments within each season.

**Treatment**	**Spring15**	**Mid15**	**Fall15**	**Spring16**	**Mid16**	**Fall16**
All crop sequences^a^	< 0.001^***^	< 0.0001^***^	< 0.001^***^	< 0.001^***^	< 0.001^***^	< 0.001^***^
Soy vs. Corn	0.45	< 0.001^***^	< 0.001^***^	0.44	< 0.001^***^	< 0.001^***^
Soy only	< 0.001^***^	0.04^∗^	0.01^∗^	< 0.001^***^	0.04^∗^	0.07.
Corn only	0.13	0.5	0.24	0.02^∗^	0.29	0.44

*^*a*^All crop sequences included comparison across all 10 crop sequences; soy vs. corn compared between all pooled treatments from each different crop; soy/corn only compared among crop sequences with different number of years of monoculture of soybean or corn grown in each year. Significant differences are reported at FDR corrected (^∗^) *P* < 0.05, (^∗∗^) *P* < 0.01, and (^∗∗∗^) *P* < 0.001.*

**FIGURE 3:**
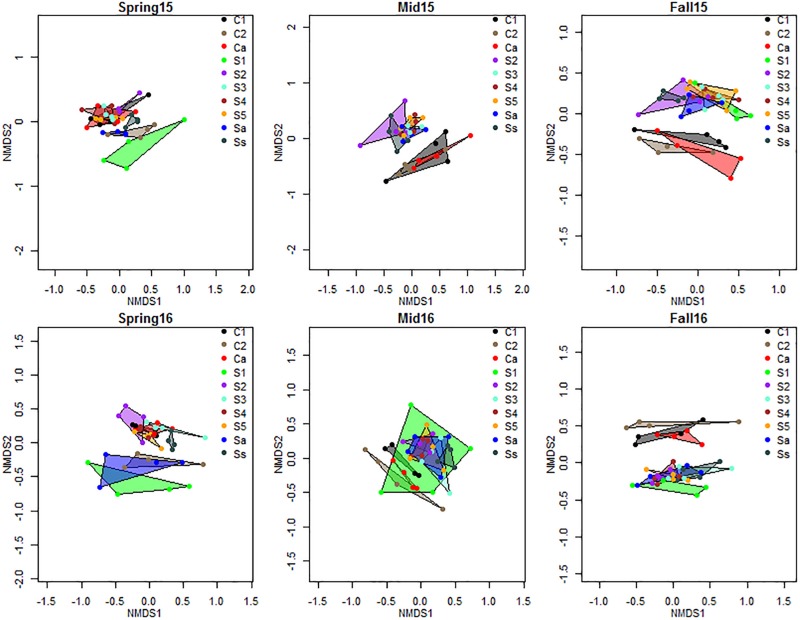
NMDS plot of Bray–Curtis distance matrix of bacterial communities by crop sequences. Crop sequence treatments include first (C1) and second (C2) year of corn following 5-year soybean rotation, annual rotation of corn (Ca) following annual rotation of soybean (Sa), years 1–5 (S1–S5) of soybean following a 5-year corn rotation, and susceptible soybean (Ss) monoculture. Color coded shapes connect the four replicate datapoints representing communities from four replicate plots of each crop sequence.

The NMDS plot showed a consistent trend of the effect of crop sequences of each crop on the bacterial communities, with differentiation between cysts from crop sequences of corn and soybean increasing over the growing season, and showing the greatest divergence in the fall of each year (Figure 3). In spring, the centroids of communities from early years of soybean (S1 and Sa) clustered more closely with the second year of corn (C2) sequence, while the remaining soybean crop sequences clustered together at a different location on the NMDS plots (Figure 3). However, by fall, the cysts associated with S1 and Sa clustered more closely with other soybean crop sequences (S2, S3, S4, S5, and Ss), than with the corn crop sequences (C1, C2, and Ca), and bacterial communities were more clearly differentiated by crop species (Figure 3). The Adonis PERMANOVA analysis provided statistical support for this trend. Although differences between cysts across all crop sequences were significant (*p* < 0.001) at all sampling seasons in both years (Table 4), when cyst communities were compared between pooled crop sequences associated with each crop species (e.g., corn and soybean), no significant differences were observed in spring, but significant differences (*p* < 0.001) were observed in midseason and fall of both years (Table 4), indicating that differences between cyst communities from crop sequences associated with each crop became more pronounced over the course of the growing season (Figure 3 and Table 4). In addition, soybean monoculture year (S1–S5, and Ss) significantly affected bacterial community structure at all sampling seasons, except in fall of 2016 (Table 4). In contrast, corn monoculture year (C1, C2, and Ca) only significantly affected bacterial community structure in spring of 2016 (Table 4). Beta-dispersion parameters, a measure of variation observed across samples of the same treatment, also varied significantly in midseason of 2015 and spring and fall of 2016 (Supplementary Table S5).

**FIGURE 4:**
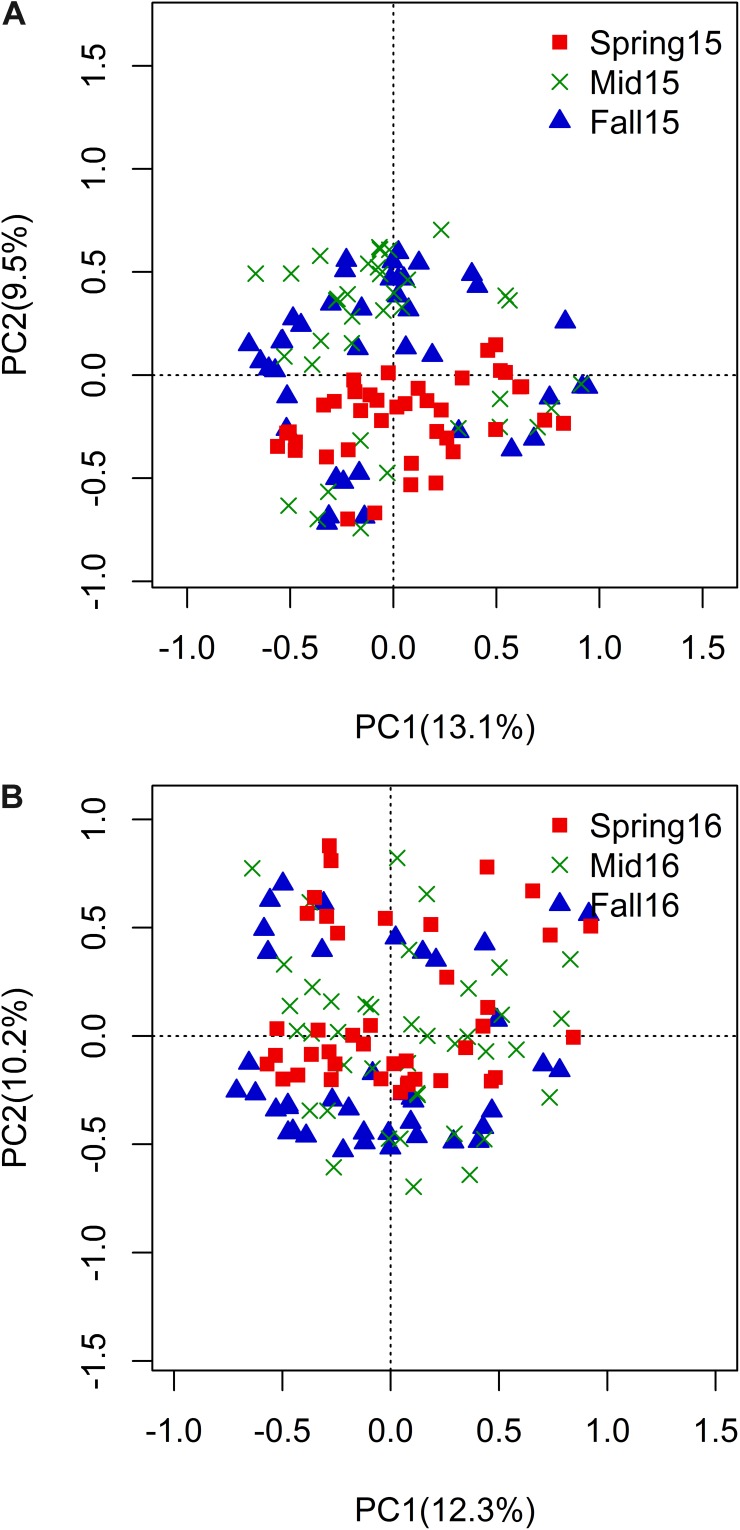
PCoA plot of Bray–Curtis distance matrix by season within year 2015 **(A)** and 2016 **(B)**. Percentage of variation explained by season was shown on PC1 and PC2. Red squares = spring, green “x” = midseason, and blue triangles = fall.

In pairwise comparisons of sampling seasons (e.g., spring vs. midseason and midseason vs. fall) within each year, the cyst bacterial communities were significantly different across all crop sequences according to the Procrustes test using the Bray–Curtis distance matrix (Figure 4 and Table 5). However, the seasonal effects were not consistently observed across all crop sequences or the same crop sequence across both years (Table 5). The most significant (*p* < 0.05) differences observed between cyst communities in comparisons between spring and fall were between early years of crop rotation following monoculture (C1 in 2015, C2 in 2016) and annual rotation (Ca and Sa in 2015, and Ca in 2016) (Table 5). Overall, the earlier years of crop rotation (C1–C2 and S1–S), as well as annual rotation (Sa and Ca), showed more differences across season, while the long-term soybean monoculture crop sequence (Ss) appeared to have a more stable community composition, with no significant (*p* < 0.05) differences observed between seasons over the growing season in either year (Table 5).

**TABLE 5:** Procrustes test of Bray–Curtis dissimilarity between seasons with FDR adjusted *P-*values < 0.1 (.), < 0.05 (^∗^), < 0.01 (^∗∗^), and < 0.001 (^∗∗∗^).

	**2015**			**2016**		
		
**Community**	**Spring vs. mid**	**Mid vs. fall**	**Fall vs. spring**	**Spring vs. mid**	**Mid vs. fall**	**Fall vs. spring**
Overall	0.001^∗∗^	0.001^∗∗^	0.001^∗∗^	0.001^∗∗^	0.001^∗∗^	0.001^∗∗^
C1	0.05^∗^	0.13	0.05^∗^	0.14	0.17	0.14
C2	0.24	0.25	0.14	0.14	0.04^∗^	0.05^∗^
Ca	0.14	0.08	0.05^∗^	0.24	0.08	0.05^∗^
S1	NA	NA	1.0	0.33	0.42	0.10
S2	0.33	0.19	0.17	0.19	0.46	0.24
S3	0.05^∗^	0.46	0.43	0.14	0.04^∗^	0.19
S4	0.86	0.67	0.1.	0.33	0.67	0.48
S5	0.57	0.25	0.24	0.05^∗^	0.08.	0.14
Sa	0.19	0.42	0.05^∗^	0.24	0.08.	0.14
Ss	0.19	0.63	0.43	0.43	0.29	0.9

### Bacterial Taxa Enriched and Associated With Cysts From Soybean Versus Corn Crop Sequences

The most significant differences of cyst bacteria between corn and soybean crop sequences were observed in fall of both years. In fall of 2015 and 2016, only about half of the observed OTUs in cysts (55.8 and 54.7% for 2015 and 2016, respectively) were shared between corn and soybean crop sequences, while 21.8 and 19.4% of cyst OTUs were unique to corn crop sequences and 22.4 and 25.9% of cyst OTUs were unique to soybean crop sequences in 2015 and 2016, respectively (Supplementary Figure S2).

**FIGURE 5:**
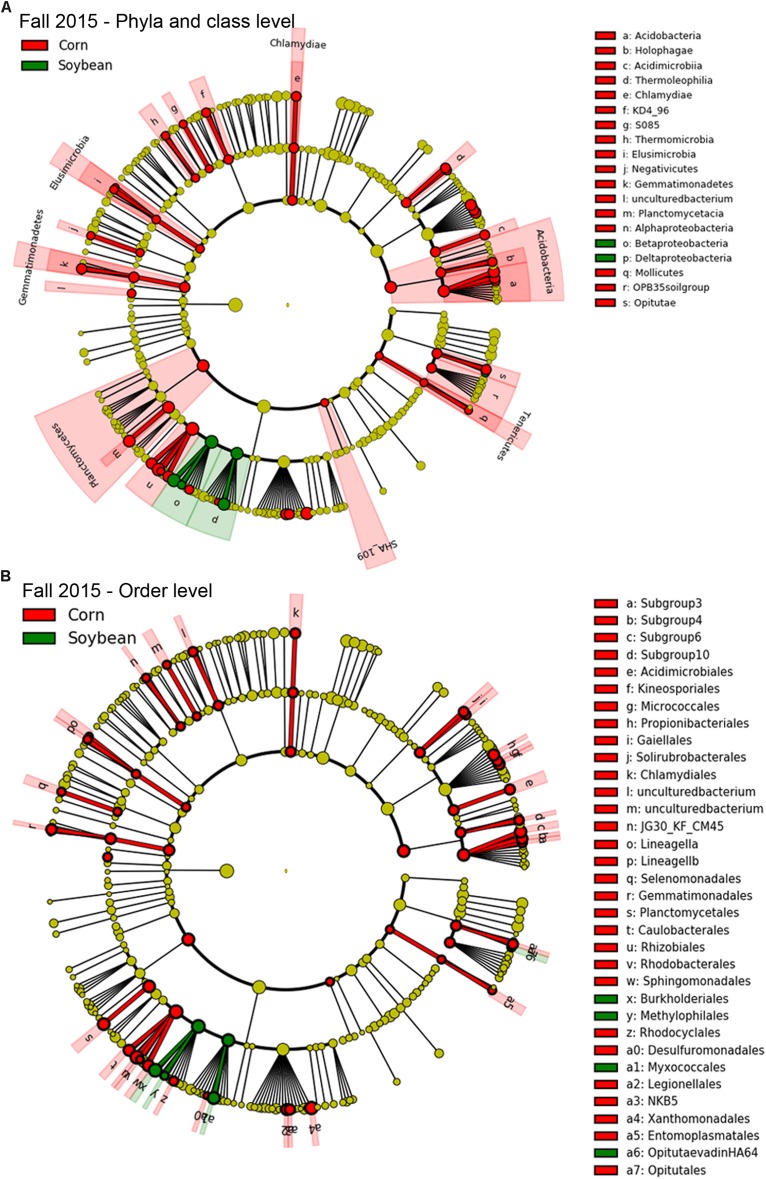
Taxa enriched in cysts of corn and soybean crop sequences identified by LEfSe analysis in fall of 2015. Cladograph shows taxa at different phylogenetic ranks that are significantly discriminant in cysts from soybean (green) and corn (red) crop sequences in fall 2015 at level of both bacterial **(A)** phyla and classes and **(B)** orders. The colored wedges indicate phyla/class and orders that are significantly discriminant at that phylogenetic rank. Letters code the specific classes or orders shown in the legend to the right. Smaller colored circles in each ring indicate significantly discriminant taxa at each lower taxonomic hierarchical level below phyum [e.g., order, family, genus, and species (OTU)] within bacterial classes. A full list of taxa used to generate this cladogram is given in Supplementary Table S6.

**FIGURE 6:**
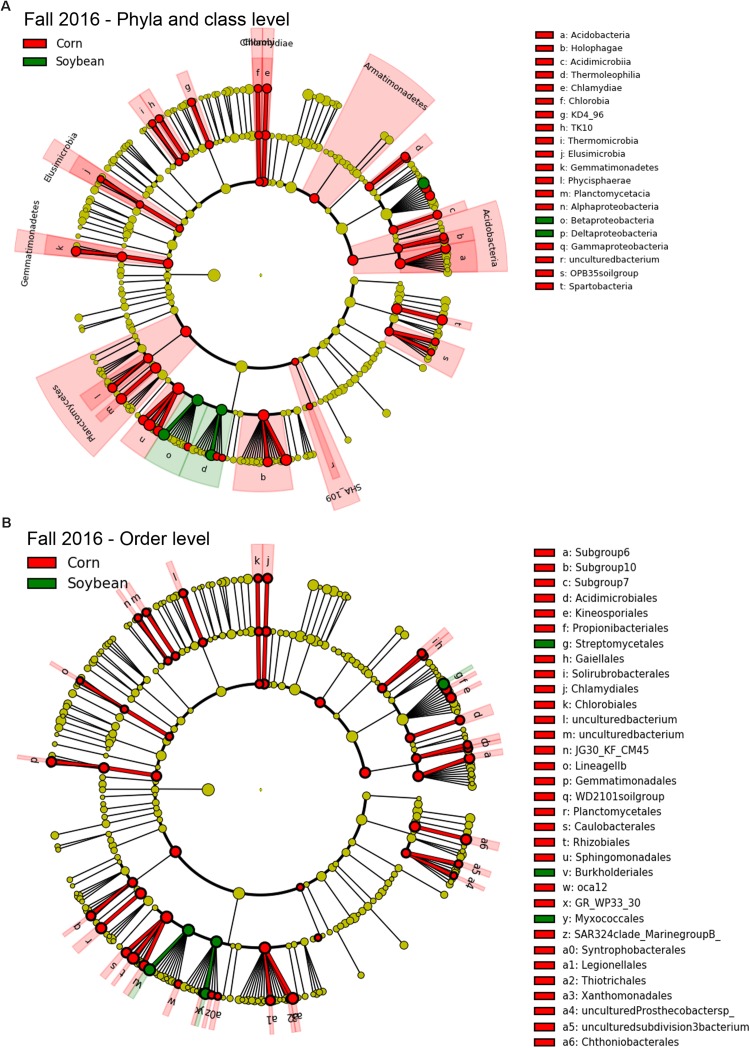
Taxa enriched in cysts of corn and soybean crop sequences identified by LEfSe analysis in fall of 2016. Cladograph shows taxa at different phylogenetic ranks that are significantly discriminant in cysts from soybean (green) and corn (red) crop sequences in fall 2016 at level of both bacterial **(A)** phyla and classes and **(B)** orders. The colored wedges indicate phyla/class and orders that are significantly discriminant at that phylogenetic rank. Letters code the specific classes or orders shown in the legend to the right. Smaller colored circles in each ring indicate significantly discriminant taxa at each lower taxonomic hierarchical level below phyum [e.g., order, family, genus, and species (OTU)] within bacterial classes. A full list of taxa used to generate this cladogram is given in Supplementary Table S6.

Since few OTUs in cysts were found to be positively correlated or to significantly increase in relative abundance with increasing years of soybean monoculture, in order to detect bacterial taxa that were significantly different in abundance between cysts from each crop species (e.g., corn and soybean), we used a class-based biomarker detection method (LEfSe) (Segata et al., 2011). This approach detected bacterial classes and orders that showed a significant difference in relative abundance in cysts between soybean versus corn crop sequences (Figures 5, 6 and Supplementary Table S6). The fall sampling time points, where the greatest differences between cyst bacterial communities between corn and soybean were observed, were chosen for this analysis. When analyzed at the class level, the Deltaproteobacteria and Betaproteobacteria showed the strongest signal for significant enrichment in cysts associated with soybean crop sequences for both 2015 (Figure 5A; green) and 2016 (Figure 6A; green). A much larger number of bacterial classes were significantly associated with cysts from corn crop sequences, with the most significant enrichment in classes Alphaproteobacteria, Planctomycetacia, Acidobacteria, followed by Gammaproteobacteria (Figures 5A, 6A; red and Supplementary Table S6). Here we focus primarily on taxa that were consistently enriched in cysts from soybean crop sequences and may have potential for biological control of the SCN. Orders contributing the most signal within the enriched classes for soybean crop sequences included the Burkholderiales within the Betaproteobacteria and Myxococcales within Deltaproteobacteria, which were enriched in both years (Figures 5B, 6B; green and Supplementary Table S6). Streptomycetales within Actinobacteria were enriched only in 2016 (Figure 6B; green) and Methylophilales within Betaproteobacteria and a member of vadinHA64 (Opitutae class of Verrucomicrobia) were enriched only in 2015 (Figure 5B; green).

A much larger number of bacterial classes and orders were found to be enriched in cysts from corn crop sequences. Classes that were significantly (*p* > 0.05) enriched in both years included several classes in phylum Acidobacteria (Acidobacteria, Holophagae) and Proteobacteria (Alphaproteobacteria and Deltaproteobacteria), as well as Classes Thermoleophilia, Chlamydiae, Elusimicrobia, Gemmatinomnadetes, Planctomycetacia, Thermomicrobia, KD4-96 group, OPB35 soil group, and the SHA_109 group (Figures 5, 6 and Supplementary Table S6). Additional classes that were enriched only in 2015 included Acidomicrobia, Betaproteobacteria, Mollicutes, Negativicutes, Opitutae, and the S085 group, while those enriched only in 2016 included Acidimicrobiia, Chlorobia, Gammaproteobacteria, Phycisphaerae, Spartobacteria, the TK10 group, and a class within phylum Armatimonadetes (Figures 5, 6 and Supplementary Table S6). Orders within these classes that were significantly enriched (corrected *p* < 0.00001) in both years included Subgroup6 (Acidobacteria), Acidimicrobiales (Acidimicrobiia), Solirubrobacterales (Thermoleophilia), Gemmatimonadales (Gemmatinomnadetes), Planctomycetales (Planctomycetacia), Rhizobiales and Rhodospirillales (Alphaproteobacteria), and Legionellales and Xanthomodales (Gammaproteobacteria).

The hierarchical structure of the LEfSe analyses also allowed us to analyze results at the genus and OTU level to detect specific OTUs within these higher level taxonomic groups enriched in cysts from soybean versus corn crop sequences. For soybean, these analyses identified taxa within several additional classes, including Cytophagia (Phylum FCB Group, Bacteroidetes), Alphaproteobacteria (Phylum Proteobacteria), and Spartobacteria (Phylum: PVC Group, Verrucombicrobia), that showed a significant association with cysts from soybean crop sequences (Supplementary Table S6). Within Burkholderiales (Betaproteobacteria), genera within the family Comamonadaceae (*Leptothrix*, *Rhizobacter*, and *Aquincola*) consistently showed the strongest signal for enrichment and association with soybean crop sequences across both years (Table 6 and Supplementary Table S6). In 2016, a symbiont of arbuscular mycorrhizal fungus (*Candidatus glomeribacter*) (Salvioli et al., 2016) within Burkholderiales was enriched in cysts from soybean crop sequences (Supplementary Table S6). Other genera that were enriched in both years included members of the Cytophagia (Bacteroidetes), including genera within the orders Cytophagales (*Cytophaga*) and Sphingobacteriales (*Chitinophaga* and *Niastella*). Within the Deltaproteobacteria, genera within order Myxococcales and family Haliangiaceae (*Helangium*) were enriched in both years (Table 6 and Supplementary Table S6). Several Actinobacteria, including taxa within genera in the orders Streptomycetales (*Streptomyces*), Streptosporangiales (*Actinomadura*), and Micromonosporales (*Plantactinospora*), also showed enrichment in soybean crop sequences, but only in 2016 (Supplementary Table S6). The Alphaproteobacteria, which showed a less significant association with soybean crop sequences, included genera within the order Sphingomonadales (*Sphingomonas*). Within the Verrucomicrobia, an OTU mapping to *Candidatus xiphinematobacter*, known as a symbiont of the nematode genus *Xiphinema* (Vandekerckhove et al., 2000), was enriched in 2016, while an unknown species within order Opitutales was enriched in 2015 (Supplementary Table S6). Fewer taxa enriched in cysts in corn crop sequences could be identified to genera. Nonetheless, several genera that were enriched in both years included members of orders Rhizobiales (*Bradyrhizobium* and *Variibacter*) and Rhodospiralles (*Reyranella*) in Alphaproteobacteria, as well as orders Legionellales (*Aquicella*), Planctomycetales (*Planctomycetes*), and Solirubrobacterales (*Solirubrobacter*) (Table 6 and Supplementary Table S6).

**TABLE 6:** Genera that were significantly (*P* < 0.05) enriched and associated with cysts from soybean and corn crop sequences in fall of both 2015 and 2016.

**Soybean**	**Corn**
**Betaproteobacteria**	**Alphaproteobacteria**

Bukholderiales	Rhizobiales
Comamonadaceae	Bradyrhizobiaceae
*Rhizobacter*	*Bradyrhizobium*
*Leptothrix*	Xanthobacteraceae
	*Variibacter*
	Rhodospirillales
	Rhodospirillales Incertae Sedis
	*Reyranella*

**Cytophagia**	**Gammaproteobacteria**

Cytophagales	Legionellales
Cytophagaceae	Coxiellaceae
*Cytophaga*	*Aquicella*

**Deltaproteobacteria**	**Planctomycetacia**

Myxococcales	Planctomycetales
Haliangiaceae	Planctomycetaceae
*Haliangium*	*Planctomyces*

**Sphingobacteria**	**Thermoleophilia**

Sphingobacteriales	Solirubrobacterales
Chitinophagaceae	Solirubrobacteraceae
*Chitinophaga*	*Solirubrobacter*
*Niastella*	

## Discussion

Because current management strategies for the SCN have significant limitations, an integrated management plan that includes use of biological control is essential for long-term and sustainable SCN management. However, only a limited number of bacterial species have been identified from cysts of the SCN based on traditional culture-based methods (Chen and Dickson, 2012), and even fewer have been tested in greenhouse or field trials or developed for their biological control potential, such as *P. nishizawae* (Clariva^TM^) and *B. firmus* (PonchoVOTiVO^TM^). Characterization of the microbial diversity of bacteria that could have activity against nematodes is useful for advancing management of this serious pathogen. This study utilized a high throughput metabarcode sequencing approach to uncover the diversity of bacterial taxa and the effects of crop host on bacterial communities directly associated with the cysts of SCN in field trials.

### Taxonomic Composition of Bacterial Communities in Cysts

Overall, the dominant bacteria in SCN cysts belonged to Proteobacteria (Alpha-, Beta-, Delta-, and Gamma-Proteobacteria) and Actinobacteria, followed by Bacteroidetes and Verrucomicrobia, which is mostly consistent with previous studies based on DGGE and culture-based approaches (Nour et al., 2003) as well as culture-independent methods (Hussain et al., 2018). A recent study found that the dominant bacteria accumulating in cysts in an SCN-suppressive soil challenged with nematodes were Proteobacteria, followed by Actinobacteria and Bacteroidetes (Hussain et al., 2018). This same study found that, in addition to these three most abundant phyla, Verrucomicrobia, Planctomycetes, Chlamydia, and Firmicutes were enriched in cysts compared to the rhizosphere or the root endosphere communities (Hussain et al., 2018).

### Changes in Bacterial Community Composition by Crop Sequence and Season

An understanding of the dynamics of microbes associated with SCN cysts in agroecosystems is also important for developing biocontrol agents that will be able to persist in commonly used crop rotation sequences. The bacterial community composition of cysts was significantly (*p* < 0.001) different when analyzed across all crops sequences according to the Adonis PERMANOVA analysis at nearly all sampling time points (Table 4), suggesting that crop sequences significantly affected bacterial communities in cysts. However, we also observed a strong interaction between crop sequences and season (Table 3), suggesting that the effect of crop sequence varied by season.

A previous companion study investigating the dynamics of fungal communities at the same field site identified several known biological control fungi whose relative abundance increased with increasing years of soybean monoculture, but did not show as strong a differentiation between corn and soybean crop sequences over a single growing season (Hu et al., 2018). In contrast, the current study revealed that the composition of bacterial communities within cysts from annual rotation (Ca, Sa) and early years of crop monoculture after 5 years of the alternate crop (C1, S1) changed more rapidly over the course of the growing season compared to fungal communities from long term monoculture (Ss). Although bacterial communities in cysts were not significantly different between corn and soybean crop sequences in spring of each year, at the end of a single growing season, they were clearly differentiated between corn and soybean crop sequences. This pattern was observed in the NMDS plot (Figure 3), and was supported statistically by the adonis PERMANOVA analysis (Table 4), where differences in communities in cysts from corn and soybean crop sequences were not significant in the spring, but became significant (*p* < 0.001) in the midseason and fall (Table 4).

Various factors could explain the more rapid changes observed in cyst bacterial communities compared to fungal communities over the course of the growing season. A faster rate of reproduction and turnover of bacteria compared to fungi in soil may be one factor. Another possible explanation is that the soybean plants may affect the bacterial communities in cysts, possibly through modifying the relative abundance of some bacterial taxa in the rhizosphere or inside roots of the crop plant that may serve as a source of inoculum and colonize newly formed cysts on the root. The hypothesis that root endophytes may infect SCN cysts forming on roots has been put forth previously for fungal parasites of nematodes (Carris et al., 1986, 1989). In support of this hypothesis, Hussain et al. (2018) reported that the bacterial community enriched in SCN cysts in a suppressive soil challenged with SCN showed more similarity with bacterial taxa enriched in the root endosphere compared to those in rhizosphere and bulk soil and also showed progressive enrichment of taxa from the rhizosphere to root and cyst communities. The use of susceptible soybean varieties in our study may also have increased the density of nematode cysts in soybean monoculture crop sequences. Some obligate parasites and pathogens of the SCN are known to show a density-dependent population dynamic in which the populations of the parasite and infection rates increase when populations of the nematode host are high (Persmark et al., 1996). Changes in other abiotic factors such as soil physio-chemical properties, rainfall, and temperature may also have contributed to changes in bacterial communities in cysts over the growing season.

### Changes in Alpha Diversity by Crop Sequence and Season

Although patterns of alpha-diversity were not entirely consistent across the two years, potentially due to differences in weather and precipitation between years, or between all crop sequences, the cyst bacterial communities in corn crop sequences tended to be more diverse, especially by the Shannon index, than those from soybean sequences (Table 2). The higher overall diversity of bacterial communities from corn crop sequences may have contributed to the larger number of taxa that were enriched in cysts from the corn crop sequences in the LEfSe analysis (Figures 5, 6 and Supplementary Table S6).

The alpha diversity of the bacterial community in cysts also changed over the crop growing season, with midseason generally showing less diversity than either spring or fall, except for observed OTUs in 2016 (Table 1). The crop sequences also had a stronger influence on alpha diversity in spring and fall (Table 2). It is possible that at midseason, bacteria that had already colonized most of the available cysts prevented other bacteria from colonizing due to antagonism. Such “priority effects,” where initial colonists inhibit new colonists is a phenomenon that has been demonstrated for fungi in SCN cysts (Chen and Chen, 2003). Between the mid and fall seasons, however, a second generation of new cysts would be produced and be available for colonization by diverse bacterial taxa. The previous study on the fungal communities in SCN cysts at this same site, for example, showed that diversity was the greatest in fall at harvest (Hu et al., 2018). It is also possible that higher temperature or other abiotic factors at midseason caused preferential proliferation of bacteria adapted to these conditions and reduced diversity between crops.

### Bacterial Taxa Enriched in Cysts From Soybean and Corn Crop Sequences

Bacterial taxa enriched in cysts from the soybean crop sequences discovered in this study provide important information for directing future biocontrol screening efforts, as one of the key characteristics of a successful biological control agent is its abundance and ability to persist in crop rotations of the affected host (e.g., soybean). The biomarker approach used in this study was also able to pinpoint more precisely specific OTUs that contributed to differences of bacterial communities in cysts between corn and soybean crop treatments (Table 6 and Supplementary Table S6), and may play an important role in SCN population management. This approach identified specific OTUs within these genera that were differentially abundant and associated with crop sequences of corn or soybean. Unfortunately, many of these OTUs could not be identified to species given current limitations in metabarcoding sequence regions and lack of representation of bacterial taxa in databases. These results suggest that identification of species or even strains that differ in abundance between crop sequences and treatments will be important to identify isolates with biocontrol potential and point to the need to improve taxonomic resolution of metabarcoding approaches for characterizing microbial communities.

Our results agree well with previous studies aimed at identifying bacterial taxa that are enriched in SCN suppressive or long-term soybean monoculture soils, but also identified some novel taxa enriched in cysts under crop sequences of each crop. Investigation of bacterial communities directly associated with control of nematodes has mostly focused on suppressive soil systems. A recent study found that several bacterial genera, including *Pseudomonas* (Gammaproteobacteria), *Burkholdaria* (Betaproteobaceria), *Chitinophaga* (Sphingobacteria), and *Streptomyces* (Actinobacteria), were enriched in the rhizosphere of soybean in long-term soybean monoculture fields that were also suppressive to SCN (Hamid et al., 2017). Another study found that when soil was amended with chitosan, a deacetylated form of chitin, taxa within *Streptomyces* increased in the rhizosphere of soybean grown in the presence of SCN and the nematode parasitic fungus *Hirsutella minnesotensis* (Mwaheb et al., 2017). Of the few studies that have investigated the effects of soybean crops sequences including long-term monoculture or SCN suppressive soils on bacterial taxa within cysts, an early study using a PCR-DGGE method and culturing approaches (Zhu et al., 2013) found that *Pseudomonas* and S*treptomyces*, as well as the nitrogen fixing rhizobacteria *Rhizobium* were enriched in cysts in a long-term soybean monoculture field. The recent greenhouse study of Hussain et al. (2018), looking at enrichment of bacterial taxa in the rhizosphere, root endosphere, and cysts in response to SCN suppressive soils and SCN challenge, found that the genera *Chitinophaga*, *Yersinia*, *Pseudoxanthomonas*, *Niastella*, *Pseudoxanthomonas*, and *Lentzea*, among others, were enriched in SCN cysts grown in SCN suppressive soil challenged with the SCN.

Plant growth promoting rhizobacteria, including several taxa within Pseudomonadales (Gammaproteobacteria) and Burkholdariales (Betaproteobacteria), are widely used in agriculture and horticulture to promote plant growth and protect plants from pathogens (Beneduzi et al., 2012; Gouda et al., 2018), including parasitic nematodes (Mhatre et al., 2019). Although we did not identify *Pseudomonas* as enriched in our study, several genera in the Burkholderiales (Betaproteobacteria) in the family Comamonadaceae (*Rhizobacter*, *Leptothrix*) were enriched in cysts from soybean crop sequences in both years (Figures 5, 6, Table 6 and Supplementary Table S6). Taxa within the genus *Rhizobacter* are intimately associated with the rhizosphere, and although to our knowledge have not been shown to play a role in nematode biocontrol, they have been shown to increase in relative abundance in a soil suppressive to a fungal wilt pathogen (Siegel-Hertz et al., 2018). *Leptothrix* was also identified in the previous study by Hussain et al. (2018) as being enriched in cysts grown in an SCN suppressive soil. It is possible that some members of Comamonadaceae could also be nematode endosymbionts, as taxa within Comamonadaceae were shown to be dominant members of the endomicrobiota of another soil invertebrate, the springtail *Orchesella cincta* (L.) (Bahrndorff et al., 2018). Surprisingly, unlike Zhu et al. (2013), we did not find enrichment of *Rhizobium* or related genera of nitrogen-fixing rhizobacteria in cysts from soybean sequences (Figures 5, 6 and Supplementary Table S6). However, the LEfSe analysis identified several OTUs belonging to *Bradyrhizobium* as enriched in cysts from corn sequences in both years (Figures 5, 6 and Supplementary Table S6). The enrichment of *Bradyrhizobium* in cysts from corn sequences was surprising. We speculate that the SCN cysts could provide an alternative habitat for these bacteria to survive in the absence of their soybean host.

In agreement with previous studies, several genera of Actinomycetes (*Streptomyces* and *Actinomadura*) were also enriched in cysts under soybean crop sequences, but only in fall of 2016 (Figure 6 and Supplementary Table S6). Taxa within *Streptomyces* have been shown in previous studies to be enriched in SCN cysts in long-term monoculture and SCN suppressive soils (Zhu et al., 2013; Hussain et al., 2018) and are well known for their production of secondary metabolite toxins or antibiotics. Some of these metabolites, including avermectins produced by the Actinomycete *Streptomyces avermitilis* (Cabrera et al., 2013) that are the active ingredient of the commercial nematicidal seed treatment Abamectin^®^, as well as others compounds may serve as nematode toxins or in antagonism of egg hatching (Liu et al., 2019). LEfSe analysis also identified OTUs within the genus *Haliangium* in the Myxococcales (Deltaproteobacteria) or “slime bacteria” to be enriched in cysts from soybean crop sequences in fall of both years (Figures 5, 6, Table 6, and Supplementary Table S6). The Myxococcales are common soil bacteria and some species are known to produce secondary metabolites with bioactivity against eukaryotes (Weissman and Muller, 2009; Mulwa et al., 2018).

The ability to parasitize SCN females and eggs requires the enzymatic machinery necessary to penetrate the outer layer of the female, which is composed mostly of collagen, as well as the egg shell, which contains chitin (Burgwyn et al., 2003). However, once the female dies and the cyst matures, bacteria can enter the cysts freely through the fenestra, the opening used for the hatched SCN juveniles to exit the cyst. Few studies, however, have tested whether the bacteria colonizing cysts are able to directly penetrate eggs or the cuticle of the nematode juveniles inside eggs and parasitize live eggs. Presumably, enzymatic mechanisms or production of toxins are the main mechanism for these cyst-colonizing bacteria to penetrate and parasitize SCN eggs. Within the Sphingobacteria (Bacteroidetes), two genera within the family Chitinophagaceae, *Chitinophaga* and *Niastella*, were enriched in cysts from soybean crop sequences in both years in this study (Figures 5, 6, Table 6, and Supplementary Table S6). Several OTUs mapping to *Chitinophaga* and *Niastella* were also found to be specifically enriched in cysts compared to rhizosphere soil and the root endosphere in SCN suppressive soil by Hussain et al. (2018). Similarly, the genus *Cytophaga* (Cytophagia) was enriched in cysts from soybean crop sequences in both years (Table 6 and Supplementary Table S6). Some species within the genus *Chitinophaga* (*Chitinophaga japonensis*) have chitinolytic abilities (Sangkhobol and Skerman, 1981; Proenca et al., 2017), while the genus *Cytophaga* includes the well-studied cellulose degrading bacterium *Cytophaga hutchinsonii*. We speculate that the ability to degrade chitin and perhaps other carbon compounds found in either the outer cyst wall or egg shell may enable colonization of SCN cysts and eggs by taxa within these genera. Future studies should investigate taxa within these genera for their ability to degrade chitin and other carbon compounds found in SCN cysts or to directly parasitize SCN eggs. Interestingly, a *Chitinophaga* sp. found as an endosymbiont of *Fusarium keratoplasticum*, a seedborne fungus, was shown to significantly alter the carbon substrate usage of its fungal host (Shaffer et al., 2017). Species of *Fusarium* have been shown to be common inhabitants of SCN cysts (Carris et al., 1989; Haarith et al., 2019) and further investigation into potential bacterial–fungal symbioses that may promote colonization of SCN cysts and parasitism of nematode eggs by both partners is a promising area of future research.

## Conclusion

This study characterized the taxonomic diversity of bacteria found within SCN cysts and showed that seasonal effects, crop sequences of corn and soybean, and the interaction of these factors shaped bacterial communities within cysts. The bacterial community structure within cysts showed a differentiation between corn and soybean over the course of a single growing season. The alpha diversity of bacterial communities in cysts was greatest in spring and fall and decreased slightly in midseason and diversity was slightly greater in corn sequences than in soybean sequences. Proteobacteria comprised a majority of the taxa found in cysts followed by Actinobacteria, Bacteroidetes, and Verrucomicrobia. A biomarker-based analysis (LEfSe) identified specific OTUs within these broader bacterial phyla that showed significantly greater abundance and a consistent association with SCN cysts under soybean cropping sequence, a desirable characteristic of an SCN biocontrol organism. Specific bacterial groups found to be enriched and associated with cysts from the soybean crop sequences included *Rhizobacter* and *Leptothrix* in the Betaproteobacteria, potential toxin producing taxa within Actinobacteria (*Streptomyces* and *Actinomadura*), *Helangium* in Deltaproteobacteria, and several genera in Bacteroidetes (*Chitinophaga* and *Cytophaga*). This study identified a diversity of bacteria found within SCN cysts that can be investigated as bacterial biological control agents of the SCN, for production of nematicidal metabolites, and for the discovery of novel chitin degrading enzymes.

## Data Availability Statement

The datasets generated for this study can be found in the NCBI accession number: PRJNA495048.

## Author Contributions

KB and SC co-conceived of and supervised the research. WH performed metabarcode sequencing of samples, designed metabarcoding analysis pipelines, analyzed the data, and co-wrote the manuscript. NS contributed data analysis pipelines and performed literature searches for classification of nematophagous fungi. DH assisted in collecting cyst samples and edited the manuscript. WH co-wrote the manuscript with KB.

## Conflict of Interest

The authors declare that the research was conducted in the absence of any commercial or financial relationships that could be construed as a potential conflict of interest.

## Supplementary Material

FIGURE S1 Comparison of percent composition at the genus level between cyst communities found in all corn crop sequences and cyst communities found in soybean crop sequences at each season. Those genera containing greater than 2% of OTUs out of total OTUs are shown, with the genera containing the largest percentage ot OTUs at the bottom of each column.

FIGURE S2 Venn diagram of OTUs shared in cysts from soybean and corn crop sequences and those unique to cysts from each crop species in **(A)** Fall 2015 and **(B)** Fall 2016.

TABLE S1 Significant differences of bacterial classes across seasons.

TABLE S2 Significant differences of bacterial classes across crop sequences at each season.

TABLE S3 Significant differences of bacterial OTUs across crop sequences at each season.

TABLE S4 Spearman rank correlation of bacteria OTUs with increasing years of soybean.

TABLE S5 Differences in Beta dispersion parameters of bacterial communities across crop sequences.

TABLE S6 LEfSe analysis results for fall 2015 and fall 2016.
