# Supplementary figures and images for: Seasonal Variation and Crop Sequences Shape the Structure of Bacterial Communities in Cysts of Soybean Cyst Nematode
Source: Front Microbiol. 2019 Nov 21;10:2671. doi: 10.3389/fmicb.2019.02671 (PMC6882411; doi:10.3389/fmicb.2019.02671)

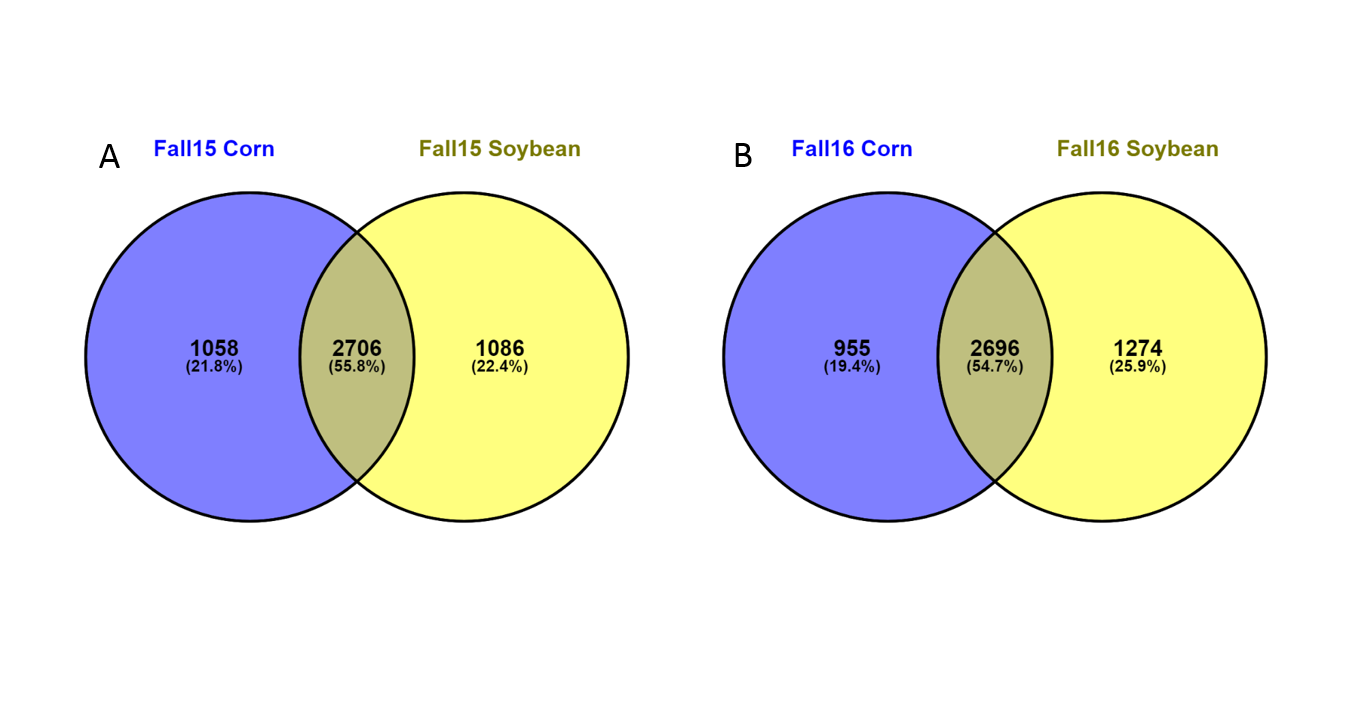

Supplement: FIGURE S2 — Venn diagram of OTUs shared in cysts from soybean and corn crop sequences and those unique to cysts from each crop species in (A) Fall 2015 and (B) Fall 2016. [file Image_2.png]
