# Supplementary material for: Seasonal Variation and Crop Sequences Shape the Structure of Bacterial Communities in Cysts of Soybean Cyst Nematode
Source: Front Microbiol. 2019 Nov 21;10:2671. doi: 10.3389/fmicb.2019.02671 (PMC6882411; doi:10.3389/fmicb.2019.02671)
Supplement: TABLE S5 — Differences in Beta dispersion parameters of bacterial communities across crop sequences. [file Table_5.DOCX]

**Table S5.** Beta dispersion parameters (centroid) of ANOVA analysis of bacterial

community across crop sequences. Significant p-values indicates by * < 0.05,

** < 0.01, and ***< 0.001.

|  |  | 2015 |  |  | 2016 |  |
| --- | --- | --- | --- | --- | --- | --- |
|  | Spring | Mid | Fall | Spring | Mid | Fall |
| Crop Seq |  |  |  |  |  |  |
| C1 | 0.37 | 0.43 | 0.38 | 0.33 | 0.35 | 0.39 |
| C2 | 0.35 | 0.36 | 0.36 | 0.40 | 0.42 | 0.41a |
| Ca | 0.37 | 0.41 | 0.42 | 0.34 | 0.36 | 0.35 |
| S1 | 0.37 | NA | 0.37 | 0.44 | 0.45 | 0.38 |
| S2 | 0.32 | 0.41 | 0.38 | 0.40 | 0.32 | 0.30 |
| S3 | 0.30 | 0.33 | 0.32 | 0.38 | 0.37 | 0.36 |
| S4 | 0.33 | 0.33 | 0.35 | 0.31 | 0.33 | 0.33 |
| S5 | 0.32 | 0.33 | 0.37 | 0.34 | 0.36 | 0.34 |
| Sa | 0.31 | 0.32 | 0.35 | 0.43 | 0.39 | 0.38 |
| Ss | 0.31 | 0.37 | 0.35 | 0.34 | 0.32 | 0.33 |
| *P* value | 0.1 | 0.02* | 0.2 | <0.001*** | 0.05. | <0.001*** |
